# PD225 Implementation Of Hospital-Based Health Technology Assessment For Innovative And Expensive Medical Devices In A French Teaching Hospital

**DOI:** 10.1017/S0266462324004446

**Published:** 2025-01-07

**Authors:** Pascale Guerre, Laure Huot

## Abstract

**Introduction:**

Teaching hospitals are the first users of innovative, and often costly, technologies. In our institution, innovative medical devices are in demand in the early stage of market diffusion, but there is little data available to help decision-makers. Although decision criteria were identified, structured and contextualized work could help the process through formalized hospital-based health technology assessment (HB-HTA).

**Methods:**

A specialist team was set up to develop an HB-HTA process dedicated to innovative and expensive devices. The process is multidisciplinary and comprises methodologists, health economists, and pharmacists. The template developed by the Adopting Hospital Based Health Technology Assessment project was used to draft the report and to identify relevant elements adapted to the local context. Literature reviews were conducted. A list of contributors was drawn up, including the medical team and the various directors involved in generating decision support information (in particular, strategic and organizational impacts). Finally, workshops were held with patient representatives.

**Results:**

Three applications have been processed, enabling the HB-HTA procedure to be improved. Coordination of the work by a dedicated independent team made it possible to establish the transparency and robustness required for an HTA process. The main difficulties were related to the multi-contributor aspect, which affected the timeline to produce the reports, introduced heterogeneity within the different items, and created some redundancy that had to be sorted out. Uncertainties were highlighted in relation to data on clinical benefits in our patient population and assumptions about activity and budget impact. The use of real-world studies to address these issues has been proposed.

**Conclusions:**

The decision-maker appreciated being able to make decisions based on a single document resulting from multidisciplinary work. The experiences were positive and have led to the creation of an HB-HTA unit. Future reports should consider the weaknesses identified, and methodological developments are underway to improve the process.

